# Concise review: breast cancer stems cells and their role in metastases

**DOI:** 10.1097/MS9.0000000000002270

**Published:** 2024-07-23

**Authors:** Mohammad Kamalabadi Farahani, Mohammad Farjadmehr, Amir Atashi, Alireza Momeni, Mahin Behzadifard

**Affiliations:** a Department of Medical Laboratory Sciences, School of Paramedical; b Department of Tissue Engineering, School of Medicine, Shahroud University of Medical Sciences; c Student Research Committee; d Department of hematology and Oncology, School of Medicine; e Department of Laboratory Sciences, School of Allied Medical Sciences, Dezful University of Medical Sciences, Dezful, Iran

**Keywords:** breast cancer stem cells, breast cancer, cancer stem cells, chemoresistance, immunophenotyping, metastasis

## Abstract

**Background:**

Breast cancer stem cells (BCSCs) have been suggested to be responsible for the development of Breast cancer (BC). The aim of this study was to evaluate BCSCs and the target organs microenvironment immunophenotyping markers in common BC metastases, and therapeutic targets regarding to the mentioned criteria.

**Material and methods:**

This narrative review involved searching international databases; PubMed, Google Scholar using predetermined keywords including breast cancer, breast cancer stem cells, breast cancer metastases, immunophenotyping, immunohistochemistry and metastases. The search results were assessed based on the title, abstract, and full text of the articles, and relevant findings were included in the review.

**Results:**

BCSCs express high amounts of aldehyde dehydrogenase 1 (ALDH1), Ganglioside 2 (GD2), CD44 and CD133 but are negative for CD24 marker. CXCR4 and OPN have high expression in the cells and may contribute in BC metastasis to the bone. Nestin, CK5, prominin-1 (CD133) markers in BCSCs have been reported to correlate with brain metastasis. High expression of CD44 in BCSCs and CXCL12 expression in the liver microenvironment may contribute to BC metastasis to the liver. Aberrantly expressed vascular cell adhesion molecule-1 (VCAM-1) that binds to collagen and elastin fibers on pulmonary parenchyma, and CXCR4 of BCSCs and CXCL12 in lung microenvironment may promote the cells homing and metastasis to lung.

**Conclusion:**

As in various types of BC metastases different markers that expressed by the cells and target organ microenvironment are responsible, BCSCs immunophenotyping can be used as target markers to predict the disease prognosis and treatment.

## Introduction

Highlights

- Breast cancer (BC) is known to be one of the most common types of cancer and the main cause of cancer-related mortality in women, which is categorized at least into five subtypes: luminal A, luminal B, human epithelial growth factor receptor type 2 (HER2), basal-like, and claudin-low
- Cancer stem cells (CSCs) have been suggested to be responsible for the development of various malignancies such as BC. BCSCs (Breast CSCs) are a small subpopulation of breast cancer cells that play a critical role in this metastasis to other organs of the body.
- BCSCs express high amounts of aldehyde dehydrogenase 1 (ALDH1), Ganglioside 2 (GD2), CD44 and CD133 but are negative for CD24 marker. CXCR4 and OPN have high expression in the cells and may contribute in BC metastasis to the bone. Nestin, CK5, prominin-1 (CD133) markers in BCSCs have been reported to correlate with brain metastasis.
- High expression of CD44 in BCSCs and CXCL12 expression in the liver microenvironment may contribute to BC metastasis to the liver. Aberrantly expressed vascular cell adhesion molecule-1 (VCAM-1) that binds to collagen and elastin fibers on pulmonary parenchyma, and CXCR4 of BCSCs and CXCL12 in lung microenvironment may promote the cells homing and metastasis to lung.

Breast cancer (BC) is known to be one of the most common types of cancer and the main cause of cancer-related mortality in women, which is categorized at least into five subtypes: luminal A, luminal B, human epithelial growth factor receptor type 2 (HER2), basal-like, and claudin-low^1,2^. Advancements in BC diagnosis and new treatment strategies that employ target therapies in combination with apoptotic ligands and chemotherapy have led to a significant decrease in the rate of the patient’s mortality^3^. Different treatment modalities have been used according to the cancer subtypes and gene expression profiles including hormonal therapies for hormone receptor-positive (HR+) ( subtypes luminal A and luminal B)^3^, inhibitors therapy for Her2-enriched BC^4^, and inhibitors of poly ADP-ribose polymerase (PARP) for triple-negative BC (TNBC) and BRCA1-mutant tumors^5^.

Despite the availability of various approaches for BC treatment, drug resistance, tumor relapse, and metastasis may occur. In such conditions, the survival rate of the patients will be very low^6^. Due to the emergence of CSCs subpopulation, drug resistance, cancer aggressiveness, and metastasis may occur because of the high tumorgenicity potential, self-renewal ability, and high invasion and migration capacity of the cells^7^. The cells are characterized by the high expression of Aldehyde dehydrogenase 1 (ALDH1), Ganglioside 2 (GD2), CD44 and CD133^8^. Additionally, Notch, Hedgehog, Wnt, Hippo, etc., signaling pathways support their stemness features^9^. In Her2-dependent BC, dissemination of certain stem cells may occur very early and even in the pre-malignant phase and the metastatic tumor cells can remain dormant in the target tissue for a long time^10,11^.

Metastases account for 90% of human cancer-related deaths^12^. BC metastasizes mainly to bone (50–65%), lungs (17%), brain (16%), liver (6%), but other organs like kidney, spleen, or uterus are the relatively rare locations^13,14^. During metastasis, certain tumor cells detach from the primary tumor, circulate in the blood, lymphatic and/or primo vascular system (PVS), and finally exit from the circulation and form a new tumor in an appropriate tissue^15^. Recurrence, metastasis, and chemo-resistant are major problems in BC patients. Metastatic tumor cells gain resistant potential that ultimately leads to failure in common therapeutic approaches including chemotherapy and radiotherapy. Several studies have focused on the molecular characteristics of the metastasis process that could help develop new therapies^16,17^. This research aims to evaluate the BCSCs and metastatic site markers that are important in BC metastases to the main site of this cancer.

## Material and methods

This narrative review involved searching PubMed and Google Scholar, using predetermined keywords, including. We conducted the PUBMED search using the following search terms: (“cancer stem cells” or “breast cancer stem cells” (“breast cancer metastases”) (“immunophenotyping” or “immunohistochemistry”) and (“microenvironment”). The search results were assessed to choose the relevant articles based on the titles and the abstracts that had including criteria. The criteria used for this review were: Breast cancer metastases; brain, bone, liver, lung, breast cancer stem cells, microenvironment, immunophenotyping, and original publications. The used exclusion criteria were articles that had not included breast cancer metastases; BCSCs; breast cancer stem cells; or microenvironment and did not assess immunophenotyping or immunohistochemistry.

## Circulating tumor cells (CTCs)

**Figure 1:**
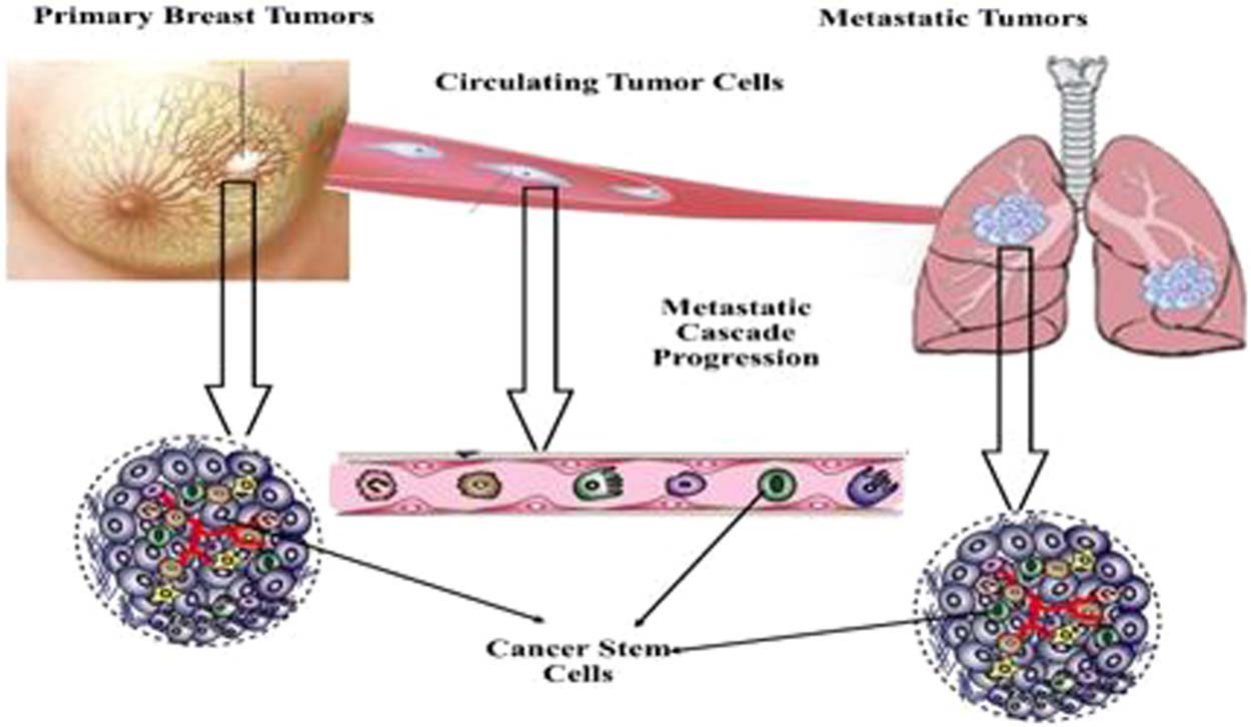
Schematic diagram depicting metastatic Breast cancer stem cells separation from initial presentation as an in-situ tumor mass at the primary site, into circulating tumor cells and induce macroscopically metastatic lesions at secondary sites.

Only asmall fraction of heterogeneous CTCs have stem cell-like and survival features and disseminate to distant organs to induce the formation of secondary tumors. These cells are known as circulating tumor stem cells (CTSCs) and usually, the detection of CTCs is associated with poor prognosis. In some patients, the cells are present with no detectable metastasis, and not all CTCs have the potency to induce metastasis. In tumor cells, reactivation of an embryonic program known as epithelial to mesenchymal transition (EMT) may occur. EMT is an important step in cancer progression that gives epithelial cells, as non-motile cells, the ability to invasion into adjacent tissues. Various cytokines originated from surrounding stroma support EMT^18,19^. CSCs are one of the actors in the EMT process and transformation into CTCs. In metastatic BC, CTCs have been identified as possible markers of metastasis correlated with worse prognosis. It is possible to distinguish mesenchymal CTCs, epithelial CTCs, and CTCs with phenotype of stemness that could have both epithelial-mesenchymal (EM) and mesenchymal-epithelial (ME) potentials. In this regard, CTCs are quite heterogeneous, and merely quantitative evaluation of t these cells cannot distinguish between prognostic values of different CTCs subpopulations (Fig. 1). Analysis of CTCs subpopulations can provide a better insight into the characteristics of CSCs. For instance, identification of EMT and stemness markers on CTCs could help identify the presence of CSCs and target the population more precisely, which are primarily responsible for disease dissemination, resistance to therapies, and ultimately worse prognosis^20,21^. ALDH1, CD44, and CD24 are known main markers associated with circulating CSCs that give the cells higher metastatic potential. Breast cancer stem cells (BCSCs) can easily switch between the ME phenotype EpCAM+CD49f+ ALDH1+, and EM phenotypeEpCAM− CD49f+ are positive for CD44/CD24. Identification of EM phenotype of circulating CSCs is based on EpCAM systems but distinguishing them from pure epithelial CTCs without further analysis is impossible^22–24^. Invasion assays for circulating subpopulations of EM-CSCs and ME-CSCs revealed that EM-CSCs had a greater invasive capacity than ME phenotype. This finding can support the notion that detected CSCs from the primary tumor undergo EMT and then enter the circulation and spread to distant organs. Before reverting to a mesenchymal/epithelial phenotype, micro-metastases are quiescent, and then CSCs could form a new bulk tumor^22,25^.

### CSCs in blood circulation

CTCs collected from metastatic BC patients usually exhibit overexpression of stem cell markers. This finding suggests that a subpopulation of CTCs that express cancer stem cell markers is responsible for induction of metastasis^25,26^. A specific subtype of CTCs can express stem cell markers CD133 and CD44 giving the cells characteristics of CSCs and then circulating cancer stem-like cells (cCSCs). The cells express CD133 and EpCAM following CD45 depletion. Notably, tumor cells expressing CD133 display CSCs features and could induce tumors in animal models^27–30^.

### CSCs in lymphatic circulation

In 80% of solid tumors, metastasis via the lymphatic system precedes metastasis via the vascular system^31^. However, the molecular characteristics of tumor cells that end up in sentinel lymph nodes (SLNs) are not fully elucidated. In a recent study using an innovative technique, lymph and lymph-circulating tumor cells (LCTCs) en route to the SLN were collected in an immunocompetent animal model of BC metastasis. Results indicated that the gene and protein expression profiles of LCTCs and blood-circulating tumor cells (BCTCs) as they exit the primary tumor were similar but distinct from those of primary tumors and lymph node metastases (LNMs)^32^. LCTCs, but not BCTCs, exist in clusters, display a hybrid EMT /CSCs-like phenotype, and are efficient metastatic precursors. These results demonstrate that tumor cells metastasizing through the lymphatic system are different from those spread by blood circulation. Understanding the relative contribution of these cells to overall peripheral blood-circulating tumor cells is essential for cancer therapy^32,33^.

## Origin of BCSCs

Current experimental evidence proposed two different but closely related theories about the origin of BCSCs and tumor heterogeneity^34^. Stochastic model or clonal evolution and Hierarchical or CSC model. The first hypothesis suggests that the tumorigenic potential of the cells residing in the tumor site is similar; however; sequential mutations in intratumoral clones can lead to tumor heterogeneity. Additionally, the origin of CSCs can be from differentiated mammary cells due to mutations that occur in the course of the disease. In this regard, the de-differentiation process leads to the generation of de novo CSCs because of exposure to radiation and/or chemotherapy agents that induce genetic alterations in non-malignant somatic cells^35^.

Hierarchical theory suggests that BCSCs arise from either mammary stem cells or progenitor cells^36,37^. This hypothesis seems more plausible because the cell surface markers such as CD44 and CD24, which are expressed on mammary differentiated progenitor cells are also expressed on BCSCs population. BCSCs display a range of abilities including self-renewal, differentiation, tumor initiation, invasion, and resistance to conventional therapy^38^.

BCSCs are characterized by a specific immunophenotype included; CD44+/CD24−, ALDH1 high, CD133+, Ganglioside 2+ (GD2+)^8,38^. CD44+CD24−/low cells have a higher ability to generate tumors upon transplantation into immune-deficient mice^39^. In ductal and inflammatory BC, ALDH1+, CD44+, and CD24− fraction enriches tumor-propagating cells and mediates metastasis and ALDH1 expression being associated with poor outcomes^40^.

It was reported that BCSCs displayed high heterogeneity among BC patients, which played a significant role in BC recurrence and metastasis, consisting of location in the tumor, biological characteristics, tumor-initiating capacity, genetic differences, and so on. Recently BCSCs have been categorized into different types, mainly according to their biomarker status, epithelial or mesenchymal status, and other biological factors. Most recently, many researches revealed that there was a potential association between BCSCs and the metastatic organotropism of breast cancer and response to treatment^41^.

## CSCs in metastatic locations

Under the physiological situation, low cell-to-matrix interaction causes anoikis that acts as a barrier and prevents cancer cells migration and metastasis. However, CSCs are resistant to anoikis and enter the circulation and grow in distant organs^42,43^. The ability to transition between mesenchymal-like (EMT) and epithelial-like (MET) cell phenotypes determines metastasis features and permits the CSCs to adapt to the changes in metastatic locations^7,44^. BC metastases may occur after years or decades of remission, and the lymph node is the preferential route of metastasis. The expression of specific molecules such as CXCR4 and CD44 on BCSCs and the presence of their ligands CXCL12, hyaluronan and osteopontin (OPN), respectively, may signify BC metastases^45,46^. OPN is involved in cell proliferation, migration, inflammation, and tumor progression in various tissues. OPN induces stemness by interacting with CD44 BCSCs express higher level of CXCR4, stimulation of CXCR4 signaling by SDF-1 induces mammosphere forming capacity and anoikis-resistance in breast cancer cells^47^. Wnt activation is also shown to be higher in the anoikis-resistant cell population^48^. Downregulation of metalloprotease-disintegrin ADAM12 reduces cell migration, invasion and anoikis-resistance in claudin-low breast cancer cells by suppressing the activation of EGFR signaling pathway^49^.

### BC metastasis to bone

Breast-to-bone metastasis (BBM) is the most frequent metastasis of BC that is predominantly diagnosed in the luminal type^50,51^. White race, young age, HR+ status, advanced tumor stage, and higher tumor grade are reported as the risk factors of BBM^52^. BCSCs reach the bone through feeder vessels of the marrow. Comparing BBM and non-bone sites, a clinical study demonstrated that 92% and 17% of cases showed high score PTHrP secretion, respectively. PTHrP leads to increased expression of the membrane protein receptor activator of nuclear factor κB (RANK) ligand (RANKL) and decreased osteoprotegerin (OPG) expression on osteoblasts membrane. RANKL promotes the differentiation of osteoclast precursors and bone matrix degradation. Osteoclasts activity leads to the release of transforming growth factor-b (TGF-b), insulin-like growth-factor-1 (IGF-1), calcium, bone morphogenetic proteins (BMPs), fibroblast growth factors (FGF) in bone environment enabling cancer cell proliferation and survival. In BBM osteolysis, hypercalcemia, severe pain, reduced mobility, and bone fractures are common, spinal cord compression and bone marrow aplasia can also occur. The enrichment of bone with OPG, TGF-b, platelet-derived growth factor (PDGF) and CXCL12 act as molecular mediators in BBM. CXCR4-CXCL12 axis has a critical role in BBM, and experimentally blocking this axis by AMD3100 (Plerixafor) led to a decrease in the BCSCs metastasis to the bone^46,51,53^. BCSCs expression of CD44, CXCR4 are high and the markers might have a role in the metastasis of BC cells to the bone (Fig. 3)^54^.

### BC metastasis to lung

BC metastasis to the lung as the second most frequent site culminates in a 5-year overall survival of 16.8%^55^. In animal experiments, it was shown that following intravenous injection of cancer cells into lung, two factors will hamper entry of cells into the lung microenvironment: first, a continuous layer of endothelial cells formed through tight cell-cell junctions, and second, killing cancer cells by leukocytes^56^. Metadherin (MTDH) is a cell surface molecule expressed by the lung endothelium and mediates BCSCs transmigration and homing to lung^57^. MTDH mediates adhesion of CSCs to the lung endothelium, thus MTDH inhibitors (the antibodies against MTDH), tyrosine kinase inhibitor (TKI); SU6668, and DNA vaccines have been suggested as inhibitors of BC metastasis to lung^56,58^. CCL2 overexpression promotes BC metastasis to both lung and bone and blocking CCL2 function with a neutralizing antibody can reduce such metastases^59^. A strong correlation of PDGF and TGF-b overexpression in BC metastasis to bone and lung was described previously^60^.

**Figure 2:**
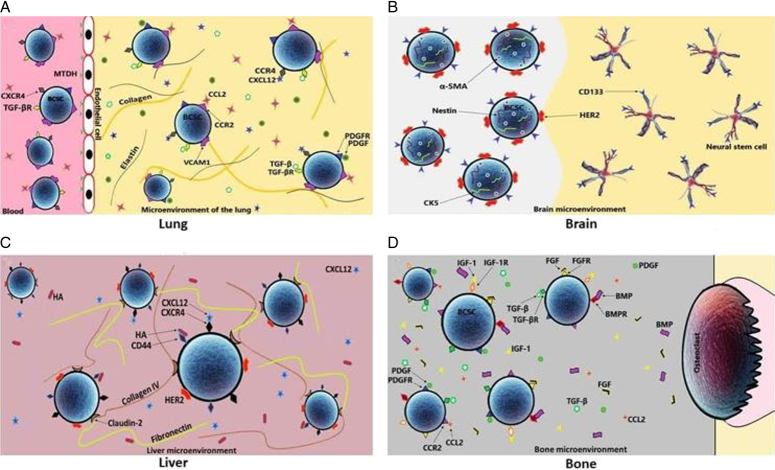
Breast cancer stem cells (BCSCs) and target organs markers in the main site of metastases, including lung (A), brain (B), liver (C) and bone (D). (A) MTDH on the surface of pulmonary vascular endothelial cells facilitates the metastasis of BCSC to the lung parenchyma. In the microenvironment of the lung, BCSCs change to CCL2 through CCR2, through VCAM-1 to collagen and elastin, through CXCR4 to CXCL12, through PDGFR to PDGF, and Through the TGF-βR on their surface, they connect to the TGF-β present in this microenvironment. (B) The expression of nestin, α-SMA, and CK5 in BCSC, as well as CD133 and HER2 on the surface of these cells, cause metastasis to the brain. (C) In the liver, BCSCs connect to CXCL12 via CCR4, to HA via CD44, and to collagen IV and fibronectin in the liver microenvironment via claudin-2. Also, the expression of HER2 on the surface of BCSCs plays a role in their metastasis to the liver. (D) In the bone, BCSCs are converted to FGF through the FGFR present on their surface, and IGF-1 through the IGF-1R on their surface, BMPR to BMP, CCR2 on their surface to CCL2, PDGFR on their surface to PDGF, and TGF-βR on their surface binds to TGF-β present in this microenvironment. α-SMA, alpha-smooth muscle actin; BMPR, bone morphogenetic protein receptor; CK5, cytokeratin 5; FGFR, fibroblast growth factor receptor; HER2, human epidermal growth factor receptor 2; IGF-1, insulin-like growth factor-1; IGF-1R, insulin-like growth factor-1 receptor; HA, hyaluronic acid; MTDH, metadherin; PDGFR, platelet-derived growth factor receptor; TGF-β, transforming growth factor-β; TGF-βR, transforming growth factor-β receptor; VCAM-1, vascular cell adhesion molecule-1.

BCSCs are not associated with luminal BC and HER2- positive subtypes, while basal-like BC (BLBC) shows a lung tropism, the primary mechanism underlying this tropism is unclear^61^. In a previous study on 4T1 mouse mammary carcinoma model, CD44vC cells showed much higher potential to promote lung colonization than CD44v cells^62^. In lung metastasis, another related study demonstrated the heterogeneity of BCSCs in clinical samples and showed that BCSCs expressed CD44v by interacting with osteopontin in the lung microenvironment promoting lung metastasis^63^. CD44-negative human BCSCs also promote lung metastasis implying that CD44 is not considered a good marker for lung invasion. BCSCs express a higher level of CXCR4 compared to normal breast tissue. Additionally, a high level of CXCR4 ligand (CXCL12) is expressed in the lung where BC cells prefer to metastasize. Increased activation of Wnt/ β-catenin signaling in BCSCs compared to normal stem-like cells, Tenascin-C (TNC), Periostin (POSTN) and Versican (VCAN) are involved in EMT and play a critical role in the BC metastasis. BCSCs aberrantly expressed vascular cell adhesion molecule-1 (VCAM-1) that bind to collagen and elastin fibers on pulmonary parenchyma and may have a role in homing and metastasis (Fig. 2)^61,64,65^.

### BC metastasis to brain

Brain is one of the target sites of BC metastases. Expression of BC brain metastases (BCBM) have a poor prognosis of around 6–18 months after the diagnosis. BC subtypes including human epidermal growth factor receptor 2 (HER2/neu)-enriched (20–30%) and TNBC (45–60%) commonly have the highest potential to induce brain metastases. Three types of BCBM, depending on the anatomic features of the brain, include parenchymal metastasis as the most common type (78%), leptomeningeal metastasis (8%), and choroid plexus are rare metastasis^66^. Compared to other metastasis sites, BCBM takes a longer time after initial BC diagnosis. This delay can be related to blood-brain barrier (BBB) with a specific compartment, including tight junctions, endothelial cells, microglia, pericytes, and astrocytes. Additionally, BBB induces a selective permeability of the brain to macromolecules. Thus, regulatory molecules and biological pathways in BBB are vital in preventing BCBM. For instance, reactive astrocytes secret miR-19a-containng exosomes which increase the aggressive metastasis of cancer cells into the brain^67^. Contribution of BBB disruption to the brain metastasis has been demonstrated not to be the case because therapeutic levels of drug conjugates were detected only in 15% of tumor lesions (Fig. 2)^68–70^.

### BC metastasis to liver

In BC liver metastasis (BCLM) the survival time is only 4–8 months. Luminal A and HER2 positive subtypes promote predominant liver metastasis compared to other BC subtypes. The liver microenvironment and sinusoidal structure have a crucial role in the initiation of homing and progression of BCLM^71^. Claudins form the backbone of the tight junction between epithelial cells and control junction permeability. Claudin-2 is weakly expressed in primary human BC. Decreased Claudin-2 mediates BCLM by enhancing adhesion to fibronectin and collagen type-IV as extracellular matrix proteins that are abundant in the liver microenvironment. It has been reported that high expression of CD44 in CSCs and CXCL12 expression in the liver microenvironment may contribute to BC metastasis to the liver (Fig. 2)^72^.

## Therapeutic targeting of breast cancer stem cell markers

Signaling pathways and surface markers that are involved in the maintenance of stemness, self-renewal and drug resistance have been proposed as therapeutic targets in BCSCs therapy. CD44, CD133, GD2, ALDH1, the main markers for BCSCs isolation, are important in BCSCs phenotypic features and maintenance^39^. Targeting the markers as potential therapeutic approaches to eradicate CSCs can be helpful. CD44 is a cell surface receptor that interacts with hyaluronic acid (HA) and is a critical molecule in the maintenance of stemness property in BCSCs. Knocking down the CD44 marker in CSCs leads to the induction of differentiation in the cells and renders BCSCs more susceptible to doxorubicin^73^. In support of the above, some studies showed that coating two anti-cancer drugs Paclitaxel and rapamycin with HA enhanced their efficacy^74,75^.

CD133 is a well-known BCSCs surface glycoprotein important in stemness maintenance, and anti-CD133-immunotoxin conjugates directly targeting CD133 molecule can be used as immunotoxin therapy for BCSCs^76^. AC-133 (a monoclonal anti-CD133 antibody) and Saponin (a known toxin) are mostly used to generate immunotoxins. AC-133 conjugation with Saponin induces cell proliferation arrest and death in CD133+ve cells. Receptor-mediated endocytosis is essential for immunotoxins delivery into the tumor cells, but since the rate of drug penetration through endocytosis and lysosomal degradation is low, immunotoxins therapy has limited efficiency^77^.

Glycosphingolipid GD2 is another BCSCs surface marker also related to stemness maintenance. Inhibition of GD2 synthesis through shRNA or small molecule triptolide can reduce CSCs population and it may be used as a therapeutic target for BCSCs eradication^78^.

**Figure 3:**
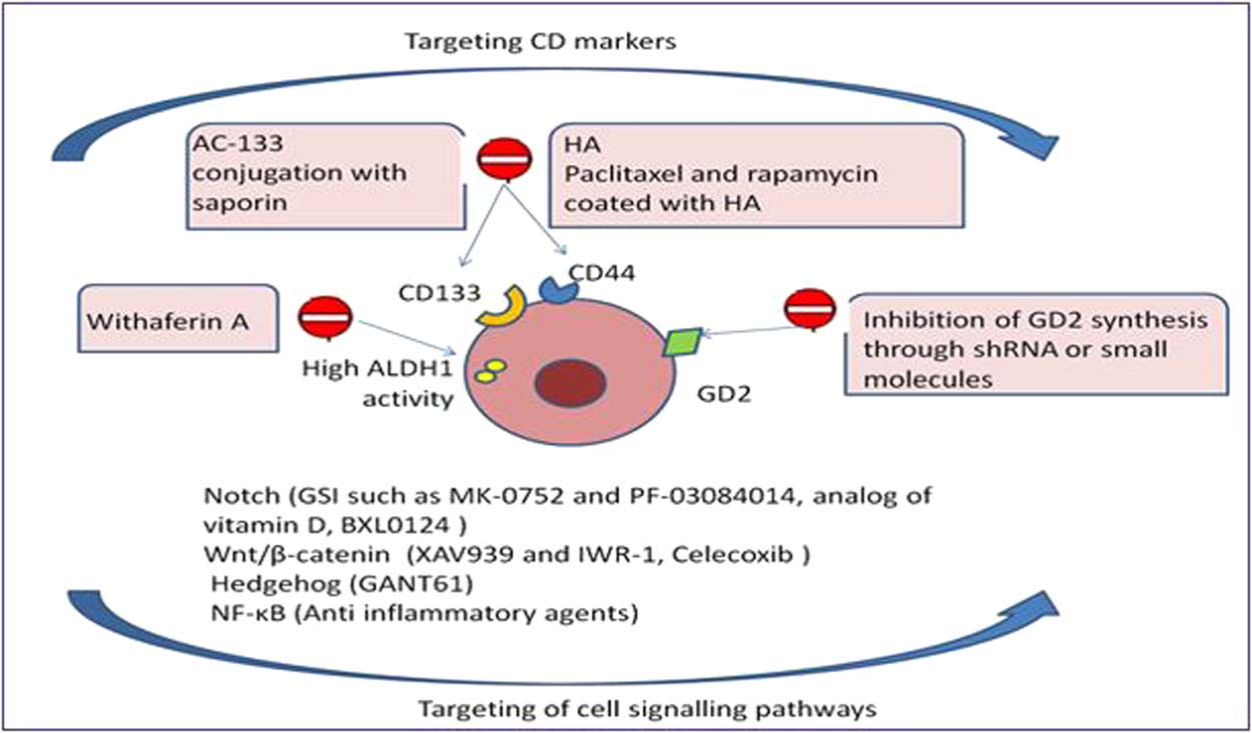
Signaling pathways and surface markers, which are involved in the maintenance of stemness, self-renewal and drug resistance can be targets in breast cancer stem cells therapy. CD44, CD133, GD2, and ALDH1 are helpful candidates for this purpose. EGFR, epidermal growth factor receptor; GSI, gamma-secretase inhibitors; HA, hyaluronic acid.

Last but not least is ALDH1. This enzyme is a phenotypic marker associated with maintaining stemness of BCSCs, and targeting ALDH1 can be used as a therapeutic approach to eradicate ALDH1+ve CSCs. Withaferin A can target ALDH1 leading to loss of BCSCs stemness^79^. In a novel method using crystallized iron oxide nanoparticles, ALDH1+ve BCSCs can be targeted and eliminated through photothermal therapy (Fig. 3)^80^.

## Targeting self-renewal pathways of breast cancer stem cells

The distinct self-renewal property of CSCs differentiates them within a heterogeneous tumor cell population. Different signaling pathways such as Notch Wnt/β-catenin, Hedgehog, Hippo, NF-κB and RTK regulate self-renewal capacity of the BCSCs^81–83^. Gamma-secretase inhibitors (GSI) such as MK-0752 and PF-03084014 can target gamma-secretase that is an important part of the notch signaling pathway in BCSCs and make them more sensitive to docetaxel^84,85^. In addition, Capsaicin could induce apoptosis in BCSCs^86^. A recent study has revealed that vitamin D compounds and Gemini analog of vitamin D, BXL0124, specifically inhibit Notch signaling pathway leading to BCSCs differentiation^87^. LGK974^88^ and Tankyrase inhibitors like XAV939 and IWR-1 have been reported to inhibit Wnt pathway, which in turn could reduce BCSC population^89^. Celecoxib is a NSAID drug that reduces BCSCs population by inhibiting the Wnt/β-catenin pathway^90^. Hedgehog pathway (HH) pathway has an important role in the regulation of stemness in BCSCs and targeting this pathway could be a helpful therapeutic approach for the eradication of CSCs population in BC. In line with this, GANT61 an inhibitor of HH can reduce the CSC population in TNBCs^91^. Trametesro biniophila murr (Huaier extract) has been shown to inhibit stemness characteristic and could reduce the BCSC population^92^. Genistein has also been used to reduce the population of BCSCs^93^. Activation of NFκB pathway has a role in the BCSCs maintenance. Different anti-inflammatory molecules such as aspirin has been shown to have a significant anti-BCSC feature^94^. Various modulators of estrogen receptor like tamoxifen or raloxifene as adjuvant therapy for hormone responsive types of BC patients could be helpful^95^. Recently EGFR has been suggested as a target for anti-BCSCs therapy, since EGFR is frequently mutated or overexpressed in different types of BC. Several anti-EGFR therapeutic agents including small molecular inhibitors and monoclonal antibodies are available (Fig. 3)^81^.

## BCSCs chemo- and radiation resistance

Radiotherapy as a standard treatment for BC patients poses complications of DNA damage due to high-energy radiation^96,97^. Furthermore, radiation resistance due to residual BCSCs has become the main challenge in BC therapy. Such resistance occurs through deranged DNA repair pathways and enhanced activity of the free radical scavenging system^98^. In breast cancer, the term inflammatory-senescence is used to describe the aging-related increase in systemic pro-inflammatory conditions in human. Inflammatory aging is a breakdown of the multi-layered cytokine network, where stem cells and stromal fibroblasts become pro-inflammatory cytokine-overexpressing cells due to the accumulation of DNA damage. Inflammatory aging is self-perpetuating because pro-inflammatory cytokines can ignite a DNA damage response in other cells surrounding DNA-damaged cells. Pro-inflammatory signals are sent by macrophages, which are key factors in the aging process of inflammation, both locally and systemically. Based on this, we hypothesize that inflamm-aging is an important factor in the increased incidence and progression of cancer in the elderly. Breast cancer is presented as a paradigmatic example of this association^99^.

The free radical scavenging capacity of BCSCs is higher than that of non-CSCs because BCSCs show higher expression of the components of the free radical scavenging system, which may decrease DNA damage and cell death mediated by reactive oxygen species (ROS)^100,101^. Radiotherapy induces the activation of NFκB^21,102^, an important transcription factor in various physiological and pathological situations^22^. NFκB activates anti-apoptotic genes including MKP1 and manganese superoxide dismutase (MnSOD), which are DNA damage scavengers and down-regulators of apoptotic signaling.^103^ In this context, the resistance of BCSCs to DNA-damaging radiotherapy may give the chance of BC aggressiveness^104,105^. Her2 as a tyrosine kinase receptor has been known as a reliable biomarker for CSCs. Her2 overexpression in CSCs is associated with tumor relapse and aggressiveness and poor prognosis^106,107^. The expression of Her2 induced by radiotherapy in BCSCs might be responsible for resistance to this therapy and increased BC aggressiveness and relapse^108^. BCSCs residing at primary and metastatic sites are responsible for intrinsic (*de novo*) drug resistance, whilst acquired (*secondary*) resistance may develop in the course of treatment^109,110^. BCSCs highly express drug efflux proteins including P-glycoprotein (ABC1), multidrug resistance-associated proteins (MRP), and breast cancer resistance protein (BCRP) that play roles in anti-cancer chemotherapy resistance^111,112^. Anti-estrogen therapy is one of the therapeutic choices for estrogen receptor-positive (ER+ve) breast cancer; however; 20–40% of ER+ve tumors acquired resistance to anti-estrogen therapy through multiple mechanisms^113^. In acquired resistance to anti-estrogen therapy, BCSCs and tumor heterogeneity play critical roles^114^. BCSCs also express a high level of ALDH1 as a detoxifying enzyme. Selective ALDH3A1 inhibition by benzimidazole analogs increases mafosfamide sensitivity in cancer cells^115^. Taken all together, targeting BCSCs drug resistance features will be helpful in controlling tumor progression and increasing patients’ disease-free survival.

## Discussion

Cancer invasion to the target organs is a complex and selective process. The proportion of CSCs among metastatic tumor cells is significantly higher than that among primary tumor cells^116^. This is partly explained by the intrinsic resistance of CSCs to anoikis, a regulated cell process that is responsible for induction of death in cells detached from the substratum. Most of the cancer cells die in the circulation whereas CSCs survive and establish metastatic lesions at distant sites^117,118^. Less than 1% of cancer cells have the chance to induce metastasis. After organ infiltration, the dormancy of CSCs determines the time of macro-metastasis. Cancer cells may not be competent to form a colony at the infiltration site. In this case, they either die or enter the dormancy phase. Dormancy is a critical potential of CSCs that adapts them to the new microenvironment and induces colonization successfully. In the dormancy stage, the cells are in the G0 phase of the cell cycle but can enter cell division in response to mitotic signals. Additionally, dormancy causes tumor cells to resist chemotherapy and the tumors relapse. CD44, CD133, GD2, ALDH1, the main markers for BCSCs isolation and are important in BCSCs phenotypic features and maintenance^39^. In this review, we aimed to summarize the current biomarkers for Cancer stem cells (CSCs) as a subpopulation of tumor cells that can drive tumor initiation and cause relapses in the metastatic process of breast cancer. We provide an overview of the most frequently used CSC markers and their implementation as biomarkers. Due to their importance, several biomarkers that characterize CSCs have been identified and correlated to diagnosis, therapy, and prognosis. However, CSCs have been shown to display high plasticity, which changes their phenotypic and functional appearance. These CSC markers might be influenced by therapeutics, such as chemo- and radiotherapy, and the tumor microenvironment. It points out, that it is crucial to identify and monitor residual CSCs. As a future perspective, a targeted immune-mediated strategy for the removal of CSCs and targeting the markers of BCSCs that are involved in maintenance of stemness, self-renewal and drug resistance have been proposed as therapeutic strategy in BC.

### Limitations of the study

We mentioned only some target therapy for BC in this review.

## Ethics approval

This study was approved by the Research Ethics Committee of Dezful University of Medical Sciences (ethical approval code: IR.DUMS.REC.1402.069).

## Consent

Not applicable.

## Source of funding

Not applicable.

## Author contribution

M.K.F. and M.B. designed the project and collected the data. A.M. and A.A. and M.F. wrote the manuscript. All authors read the final manuscript and approved it. The authors read and approved the final manuscript.

## Conflicts of interest disclosure

The authors declare no conflict of interest.

## Research registration unique identifying number (UIN)

Not applicable.

## Guarantor

Mahin Behzadifard.

## Data availability statement

Not applicable.

## Provenance and peer review

The authors declare that they have no conflict of interest to the publication of this article. The manuscript has been seen and approved by all authors and is not under active consideration for publication. It has neither been accepted for publication nor published in another journal fully or partially. Corresponding author confirm the proof of the manuscript before online publishing.
